# Case Studies From India: Management of Diabetes Using Continuous Glucose Monitoring

**DOI:** 10.7759/cureus.93177

**Published:** 2025-09-25

**Authors:** Ajay Kumar Gupta, Ajay Krishnan, Manu Kataria, Nishesh Jain

**Affiliations:** 1 Internal Medicine, Madhumeha Diabetes Clinic, New Delhi, IND; 2 Internal Medicine, Max Super Speciality Hospital, Vaishali, Ghaziabad, IND

**Keywords:** continuous glucose monitoring, diabetes in pregnancy, diabetes management, gestational diabetes, type 2 diabetes

## Abstract

Continuous glucose monitoring (CGM) has significantly advanced diabetes management by enabling real-time glucose tracking. However, many patients with diabetes still fail to achieve optimal glycemic control. This case series reviews the role of CGM by analyzing case studies from various clinical departments of a quaternary care center in Northern India. We identified four cases: a 46-year-old male patient with chronic liver disease on CGM who achieved optimal glycemic control (average glucose, 105 mg/dL; estimated glycated hemoglobin (HbA1c), 5.8%) by maintaining a time in range (TIR) of 98%; a 31-year-old female patient diagnosed with gestational diabetes mellitus (GDM) who maintained excellent glycemic control (estimated HbA1c, 6%; TIR, 96%) using basal insulin, with minimal glucose variability and no significant complications; a 28-year-old female patient with diabetes in pregnancy (DIP) who achieved a 100% TIR for over 14 days, with an average glucose of 106 mg/dL and estimated HbA1c of 5.8%. The patient was on insulin Degludec (Tresiba^®^, Novo Nordisk A/S, Bagsværd, Denmark) and experienced two minor low-glucose events but no major complications; a 69-year-old male patient with diabetes, hypertension, and coronary artery disease who had a TIR of 56% on Tresiba^®^ and oral medications, with an average glucose of 177 mg/dL and estimated HbA1c 7.5%, controlled glucose variability, and no hypoglycemic events. These cases highlight the effectiveness of CGM in managing diabetes across diverse clinical conditions in adults. The CGM system enables tailoring of treatment regimens to improve glycemic control and reduce the risk of hypoglycemia, all of which are essential for optimizing patient outcomes.

## Introduction

Diabetes mellitus (DM) is a chronic condition, with the global prevalence in 2019 estimated to be 9.3% (463 million), rising to 10.2% (578 million) by 2030 [1]. India had an estimated 74.9 million people living with diabetes in 2021, which is projected to rise to 124.9 million by 2045. According to the International Diabetes Federation (IDF), one in every seven adults with diabetes globally resides in India, and nearly one in three households in the country is affected by the condition [2,3]. Despite several advancements in glucose monitoring, many patients with DM still fail to achieve optimal glycemic control [4].

Continuous glucose monitoring (CGM) has revolutionized diabetes management by offering real-time glucose monitoring and complementing traditional blood glucose monitoring (BGM), which requires routine finger pricks. The CGM system has empowered healthcare providers, patients, and caregivers to manage diabetes more effectively through timely, data-driven decisions [5]. Moreover, compared with standard glucose metrics, such as glycated hemoglobin (HbA1c), CGM metrics, including time in range (TIR), time above range (TAR), and time below range (TBR), provide a more individualized glycemic profile [6,7]. The TIR refers to the percentage of time glucose levels are within the target range (70-80 mg/dL), while TBR indicates the percentage of time when blood glucose is <70 mg/dL, and TAR represents the percentage of time when blood glucose is >180 mg/dL. The primary goal of effective and safe glucose control is to increase the TIR while minimizing TBR [8].

Previous studies have demonstrated that in insulin-treated patients with type 2 diabetes mellitus (T2DM), the use of real-time CGM (rtCGM) can significantly improve glycemic control [9-11]. A meta-analysis involving 112 patients with T2DM reported a significant reduction in HbA1c levels with CGM compared with self-monitoring of blood glucose (mean difference, -0.31%; 95% confidence interval (CI) -0.6% to -0.02%) [10]. A recent study by Lind et al. demonstrated that the use of CGM in insulin-treated patients with T2DM led to significantly greater improvements in HbA1c and TIR compared with BGM [12]. Furthermore, Cox et al. reported that patients in the glycemic excursion minimization plus rtCGM group showed greater improvements in HbA1c compared with those in the routine care group [13].

Gestational diabetes mellitus (GDM) is one of the most common medical complications of pregnancy and is defined as glucose intolerance with onset or first recognition during pregnancy. It is typically seen between weeks 24 and 28 of pregnancy [14,15]. Unlike GDM, DIP refers to cases where diagnostic criteria for diabetes are met during pregnancy itself, indicating a pre-existing diabetes [16,17]. The prevalence of GDM also appears to be rising, though estimates vary widely across India [18,19]. According to the International Diabetes Federation, one out of six live births worldwide is diagnosed with GDM [20]. GDM is associated with an increased risk of short-term pregnancy complications, such as hypertension, preeclampsia, the necessity for a cesarean section (C-section) delivery, and difficulty during childbirth, along with an increased risk of T2DM development in mothers in the future [21,22]. Furthermore, congenital malformations, preterm delivery, neonatal hypoglycemia, macrosomia, shoulder dystocia, hyperbilirubinemia, birth trauma, and perinatal death could be other maternal-fetal and neonatal complications in women with GDM [21,23-25].

The scope of CGM has also expanded to include diabetes during pregnancy, in which strict glycemic control is critical for both the mother and the fetus [26-28]. A case report described a 31-year-old woman who developed GDM at six weeks in two consecutive pregnancies, with the first ending in spontaneous abortion at 10 weeks. In her second pregnancy, early insulin therapy led to a successful outcome, highlighting the importance of screening for GDM during the initial antenatal visit in high-risk individuals [29]. A study involving 50 insulin-treated patients with GDM observed that CGM was beneficial in this population, demonstrating improved HbA1c levels compared with standard antenatal care without increasing severe hypoglycemia [30]. CGM can safely and effectively monitor blood glucose fluctuations in patients with gestational hyperglycemia, thereby reducing adverse pregnancy outcomes [26]. 

The FreeStyle Libre Pro System (Abbott Diabetes Care Inc., Alameda, CA) has been approved for marketing in India since March 2015. FreeStyle Libre® and FreeStyle Libre Pro® have enabled adults with type 1 diabetes mellitus (T1DM) or T2DM to achieve effective glycemic control [31]. However, despite these advancements, several challenges persist in India, including high costs, limited availability, health system constraints, inconsistent adoption, and limited access for specific patient populations [32,33].

This case series aims to critically explore the role of CGM in improving glycemic control and health outcomes among patients from various clinical departments. The goal was to establish a system that supports optimal blood sugar management, thereby ensuring a safe and complication-free perioperative period. All CGM devices included had a fixed monitoring duration of 14 days. At the time of the study, no CGM device was available that extended beyond these 14 days. Additionally, the study is focused solely on monitoring blood glucose levels using these devices and does not involve any diagnostic interpretation or modification of existing diagnoses. The goal is to enhance patient comfort and provide more robust monitoring compared to traditional point-of-care finger-prick methods. A case series approach was selected to provide detailed, context-rich insights into individual patient experiences, which are often underrepresented in large-scale studies. This study presents four detailed case reports from a quaternary care center in northern India, where most patients belong to a higher socioeconomic status and possess adequate literacy, thereby highlighting the clinical utility, challenges, and benefits of CGM in varied diabetic populations.

## Case presentation

Case report 1: Management of diabetes in a 46-year-old male patient following liver transplantation

This case report details the diabetes management of a 46-year-old male patient with chronic liver disease (CLD) secondary to hepatitis B and hepatitis D who underwent liver transplantation. Without a documented duration of diabetes or use of oral hypoglycemic agents or insulin, he achieved excellent glycemic control. Fourteen-day period CGM report showed an average glucose level of 105 mg/dL, a glucose management indicator (GMI) of 5.8% (40 mmol/mol), and a TIR of 98%. Tailored lifestyle management and continuous monitoring enabled optimal glycemic control, emphasizing the importance of individualized care in complex medical conditions.

Case Presentation

Patient information: A 46-year-old male patient presented for routine follow-up. He had no documented history of diabetes and was not receiving any oral hypoglycemic agents. The patient had CLD, and his body mass index (BMI) was unavailable. The lab HbA1c was 5%.

Medical history and comorbidities: The patient had a history of CLD secondary to hepatitis B and hepatitis D. There was no documentation of other comorbidities such as hypertension, dyslipidemia, cardiovascular disease (CVD), or diabetes-related complications. The patient was stable at presentation, with no active or resolved infections following transplantation.

Clinical findings and laboratory results: The patient’s laboratory results showed an HbA1c of 5%, with an estimated HbA1c of 5.8% (40 mmol/mol). Fourteen-day period CGM data revealed an average glucose level of 105 mg/dL, with a GMI of 5.8%, glucose variability of 22.3%, and a TIR of 98%. TBR and TAR were both 1%. The sensor was active 88% of the time over the past 14 days, indicating consistent monitoring. Multiple low-glucose events were observed, on September 8, 2024, 1 event (glucose: 66 mg/dL) occurred; on September 11, 2024, 2 events (maximum glucose: 69 and 71 mg/dL; minimum glucose: 69 and 68 mg/dL) occurred; and on September 17, 2024, 1 more event (glucose: 67 mg/dL) occurred. The average duration of these low-glucose events was 241 minutes, suggesting periods of prolonged hypoglycemia, despite overall good glycemic control (Table 1).

**Table 1:** Clinical findings and laboratory results. GMI, glucose management indicator; mmol: millimole; TAR, time above range; TBR, time below range; TIR, time in range

Parameter	Target range	Case report 1	Case report 2	Case report 3	Case report 4
Average blood glucose (mg/dL)	<154 [34]	105	114	106	177
TIR (%)	>70% [8]	98%	96%	100%	56%
TBR (%)	<4% [8]	1%	3%	0%	0%
TAR (%)	<25% [8]	1%	1%	0%	44%
Glucose variability (%)	≤36%[8]	22.3%	23.30%	15.60%	18.8%
GMI (%)	<7% [34]	5.8% (40 mmol/mol)	6% (42 mmol/mol)	5.8% (40 mmol/mol)	7.5% (59 mmol/mol)
Sensor data (time active) (%)	≥70% [8]	88%	73%	94%	92%
Low-glucose events (mg/dL)	-	8/09/2024: 66	11/03/2024: 43	20/06/2024: 67 and 63	-
		11/09/2024: 69 and 71 (Max)	18/03/2024: 53 (Min)	26/06/2024: 61	-
		11/09/2024: 69 and 68 (Min)	18/03/2024: 69 (Max)	-	-
		17/09/2024: 67	-	-	-
Duration of low-glucose events (average) (minutes)	<15 [8]	241	202	45	NA

Management and Outcome

Treatment: The patient was not on any anti-diabetic medications, including metformin, sulfonylureas, dipeptidyl peptidase-4 (DPP-4) inhibitors, glucagon-like peptide-1 (GLP-1) receptor agonists, sodium-glucose co-transporter-2 (SGLT2) inhibitors, or insulin. The patient was managed without medication, through lifestyle adjustments and close monitoring after transplantation.

Outcome: The data on the patient’s glucose monitoring and TIR recorded instances of hypoglycemia, with multiple low-glucose events observed. The patient maintained optimal glycemic control through close and continuous monitoring

This case highlights the successful management of diabetes in a post-liver transplant patient without the use of insulin or oral antidiabetic agents. Through CGM and likely lifestyle interventions, the patient achieved glycemic control, demonstrating the effectiveness of a personalized management plan. Continued monitoring and tailored care are essential to address hypoglycemic episodes and ensure long-term success.

Case report 2: Management of GDM in a 31-year-old female patient

This case report outlines the management of a 31-year-old female patient diagnosed with GDM during pregnancy. Insulin therapy with a basal insulin analog was used, and the patient demonstrated excellent glycemic control throughout the pregnancy, with minimal glucose variability and no significant complications.

Case Presentation

Patient information: A 31-year-old female patient presented with GDM during pregnancy, with no previous history of diabetes or significant comorbidities. The patients BMI reported was 21.9 kg/m^2^.

Medical history and comorbidities: The patient had no records of hypertension, dyslipidemia, CVD, or diabetes-related complications.

Clinical findings and laboratory results: The average glucose level of the patient was 114 mg/dL and estimated HbA1c was 6% (42 mmol/mol), indicating good glycemic control. The patient achieved a TIR of 96%, with a TBR of 3% and a TAR of 1%. Moderate glucose variability was noted at 23.3%. The glucose monitoring sensor was active 73% of the time. Notably, several low-glucose events were recorded, including a significant episode on March 11, 2024, with a minimum level of 43 mg/dL, and multiple events on March 18, 2024, ranging from 53 to 69 mg/dL. The average duration of these low-glucose events was approximately 202 minutes. The patient’s clinical findings and laboratory results are summarized in Table 1.

Management and Outcome

Treatment: The patient's glucose levels were managed with Tresiba® (Novo Nordisk A/S, Bagsværd, Denmark), a long-acting basal insulin and metformin. The patient also received education on self-monitoring, dietary modifications, and lifestyle changes to effectively manage diabetes.

Outcome: The patient achieved excellent glycemic control, maintaining a TIR of 96% during the monitoring period. Despite a glucose variability of 23.3%, no severe hyperglycemic events occurred, and low-glucose events were effectively managed. There were no infections or complications throughout the pregnancy.

This case highlights the significance of early detection and personalized management of GDM through insulin therapy. Although some low-glucose events were observed, the overall glycemic management was successful, with minimal fluctuations and high TIR. The combination of insulin therapy and lifestyle modifications proved effective in managing GDM, thereby reducing the risk of adverse pregnancy outcomes.

Case report 3: Management of DIP in a 28-year-old female patient

This case report presents the management of a 28-year-old female patient diagnosed with DIP. The patient was initiated on insulin therapy using Tresiba®, which contributed to maintaining optimal glycemic control.

Case Presentation

Patient information: The patient was a 28-year-old female with a two-year history of diabetes. The patient’s BMI was 26.6 kg/m^2^, and her laboratory-reported HbA1c was 6.1%.

Medical history and comorbidities: The patient did not have any documented comorbidities such as hypertension, dyslipidemia, CVD, or neuropathy.

Clinical findings and laboratory results: The patient demonstrated exceptional glycemic control throughout the treatment period, maintaining an average glucose level of 106 mg/dL. Fourteen-day CGM report showed a TIR of 100% with an estimated HbA1c of 5.8%. The GMI was 5.8%, indicating well-managed blood glucose levels. Glucose variability remained low at 15.6%, and sensor activity was recorded for 94% of the time. Two minor low-glucose events were recorded, with glucose levels briefly decreasing to 67 mg/dL and 63 mg/dL on June 20, 2024, and 61 mg/dL on June 26, 2024, lasting an average of 45 minutes each. The patient’s clinical findings and laboratory results are summarized in Table 1.

Management and Outcome

Treatment: The patient was treated with Tresiba®, chosen for its stable and long-acting properties. No oral antidiabetic agents, such as metformin or sulfonylureas, were prescribed. Dietary and lifestyle adjustments were emphasized, and the patient was educated on self-monitoring of blood glucose.

Outcome: The patient achieved a TIR of 100% with no time spent below or above the target range. Glucose variability was well controlled at 15.6%. The patient experienced two isolated low-glucose events, which were effectively managed without significant complications.

This case demonstrates effective management of DIP with Tresiba®, achieving excellent glycemic control, minimal glucose variability, and a TIR. The patient had no reports of infections or complications throughout the period, highlighting the importance of individualized insulin therapy and CGM in achieving optimal maternal and fetal outcomes.

Case report 4: Management of T2DM in a 69-year-old male patient

This case study reports the management of a 69-year-old male patient diagnosed with T2DM who underwent total knee replacement and presented with multiple comorbidities.

Case Presentation

Patient information: The patient was a 69-year-old male with a history of diabetes for one year and six months. At presentation, his BMI was 34 kg/m^2^, and his laboratory-reported HbA1c was 9.9%.

Medical history and comorbidities: The patient presented with a history of previous comorbidities, including hypertension, hypothyroidism, and CAD, following coronary artery bypass graft (CABG) surgery and total knee replacement.

Clinical findings and laboratory results: The average glucose level of the patient was 177 mg/dL and had an estimated HbA1c of 7.5%. Fourteen-day CGM reports showed a TIR of 56% with no TBR and a TAR of 44%. The GMI was 7.5%, which was higher than the required goal of 7.0%. Glucose variability remained low at 18.8%, and sensor activity was recorded for 92% of the time. No low-glucose events were recorded. The patient’s clinical findings and laboratory results are summarized in Table 1.

Management and Outcome

Treatment: The patient was treated with Tresiba®, and other oral antidiabetic medications such as metformin, a DPP-4 inhibitor, and an SGLT-2 inhibitor were also prescribed. Dietary and lifestyle adjustments were emphasized, and the patient was educated on self-monitoring of blood glucose.

Outcome: Over a 14-day CGM period, the patient achieved a TIR of 56% with no TBR and TAR of 44%. The glucose variability was well controlled at 18.8%. The patient did not experience any low-glucose events.

This case demonstrates the effective management of T2DM with Tresiba® and oral antidiabetic agents, achieving significant improvement in glycemic control, minimal glucose variability, and 0% TBR. The patient had no significant complications, emphasizing the importance of individualized insulin therapy and CGM in achieving optimal outcomes.

## Discussion

Diabetes is a major global health concern, with its prevalence rising at an alarming rate. Traditionally, BGM was the standard method for individuals to check their blood sugar levels at home. However, the burden associated with frequent finger-prick testing and poor adherence limits its effectiveness, resulting in many patients not monitoring their blood glucose as frequently as necessary. Therefore, there is a growing focus on CGM technology, which has been shown to markedly decrease the risk of hypoglycemia related to treatment [35]. In this study, we present four distinct and complex cases involving surgical patients, with the primary aim of developing a system that ensures optimal glycemic control and a smooth, event-free perioperative period.

Disturbances in glucose metabolism are frequently observed after liver transplantation, with significant variability noted in the metabolic states of recipients over time. A study involving 429 adult liver transplant recipients reported that the prevalence of prediabetes was approximately 39%, whereas post-transplant DM (PTDM) was observed in approximately 21% of patients. The findings highlight the significance of routine screening for glucose metabolism disturbances in liver transplant recipients [36]. A recent study has shown that CGM is a reliable method for glucose monitoring, demonstrating superior predictive capabilities for PTDM compared with capillary BGM. In addition, the study revealed that postoperative TAR of 180 mg/dL is a risk factor associated with PTDM detected using CGM [37]. A case series by Unger and Franco emphasizes that patients with chronic diseases should be treated with a focus on achieving successful outcomes rather than allowing treatment failure. The study highlights the need for timely and proactive management strategies to support patient health and well-being. The integration of CGM into primary care settings plays a crucial role in this approach, enabling both patients and healthcare providers to reduce the risk of treatment-emergent hypoglycemia and enhance the overall quality of life for patients [35].

GDM affects 14% of pregnancies worldwide, and up to 50% of these patients will develop GDM in future pregnancies and/or T2DM later in life [38,39]. Data from studies on Asian pregnant women with overweight or obesity demonstrated CGM's potential clinical significance in the first trimester for predicting GDM and adverse pregnancy outcomes [40]. Compared to self-monitored blood glucose (SMBG), CGM offers more sensitive results in detecting hypoglycemic episodes, which may improve maternal and fetal outcomes [41]. A study on 340 women with GDM reported that the CGM group had a lower incidence of preeclampsia, primary C-section, and preterm delivery, as well as better fetal outcomes, compared with the SMBG group [42]. In a prospective study, patient compliance in the CGM group was as high as 90% [43]. Therefore, CGM is recommended for patients with DIP [44]. CGM has shown promising benefits in managing diabetes during pregnancy, improving maternal and fetal outcomes.

Older adults undergoing surgery represent a distinct subpopulation characterized by clinical heterogeneity, unique care priorities, and the integration of technology into their treatment. Although scientific evidence and clinical experience with diabetes technologies, such as CGM, are growing, their application remains limited. Few studies show CGM as a promising tool for older adults, especially those with T1DM, by helping to predict and reduce the risk of hypoglycemia and improve glycemic control [45]. A trial involving patients with T1DM aged ≥60 years showed that the use of CGM led to a significant reduction in time spent with hypoglycemia compared with periodic finger-stick monitoring using standard BGM [46]. The DIAMOND trial (clinicaltrials.gov Identifier: NCT02282397), involving participants with diabetes aged >60 years who were using multiple daily insulin injections, assigned participants to either CGM or SMBG. The use of CGM resulted in significant reductions in HbA1c and glycemic variability, time spent >250 mg/dL, and an increase in TIR. In addition, the CGM group reported high satisfaction with the device [47]. The WISDM trial randomized individuals with T1DM aged >60 years to either CGM or BGM, reporting a reduction in hypoglycemic events without increasing hyperglycemia, thus providing further evidence for fully integrating CGM into clinical practice [48]. Overall, CGM has proven to be a valuable tool for high-risk patients as well, significantly reducing hypoglycemia and improving glycemic control, with high patient satisfaction and strong clinical support for its integration into routine care.

The cases emphasize that CGM is effective in glycemic management during the perioperative period by offering a more comprehensive and real-time evaluation of glucose trends, thereby minimizing the risk of complications linked to glycemic fluctuations. Although this study provides valuable insights, it has a few limitations, including the small sample size, the lack of a control group, and limited generalizability. To maximize the benefits of CGM, it is crucial to consider that individual goals should be tailored based on each patient's unique characteristics and risk profiles. This personalized approach ensures that patients receive optimal care and support in effectively managing their diabetes [49,50].

## Conclusions

CGM is emerging as a supportive tool in the diagnosis and management of diabetes in India, especially in high-risk patients. Each of the four cases presented here from the Northern Indian population indicated that CGM is feasible, acceptable, and well-tolerated among patients from different clinical departments. This supports its role in providing a more comprehensive and robust assessment of glycemic patterns compared to traditional monitoring methods. However, the present findings are limited by the sample size, absence of control groups, and short follow-up duration, all of which constrain the generalizability of the study. Further large-scale, well-controlled, long-term follow-up studies are essential to determine whether extended use of the FreeStyle Libre® and FreeStyle Libre Pro® systems can lead to improved glycemic control and better clinical outcomes.
